# New target volume delineation and PTV strategies to further personalise radiotherapy

**DOI:** 10.1088/1361-6560/abe029

**Published:** 2021-02-25

**Authors:** David Bernstein, Alexandra Taylor, Simeon Nill, Uwe Oelfke

**Affiliations:** 1 Joint Department of Physics, The Institute of Cancer Research and The Royal Marsden NHS Foundation Trust, Fulham Road, London, SW3 6JJ, United Kingdom; 2 Gynaecology Unit, Royal Marsden NHS Foundation Trust, Fulham Road, London, SW3 6JJ, United Kingdom; 3 Joint Department of Physics, The Institute of Cancer Research and The Royal Marsden NHS Foundation Trust, London, SM2 5PT, United Kingdom

**Keywords:** target delineation, delineation uncertainty, PTV margin, geometric uncertainties, personalised medicine

## Abstract

Target volume delineation uncertainty (DU) is arguably one of the largest geometric uncertainties in radiotherapy that are accounted for using planning target volume (PTV) margins. Geometrical uncertainties are typically derived from a limited sample of patients. Consequently, the resultant margins are not tailored to individual patients. Furthermore, standard PTVs cannot account for arbitrary anisotropic extensions of the target volume originating from DU. We address these limitations by developing a method to measure DU for each patient by a single clinician. This information is then used to produce PTVs that account for each patient’s unique DU, including any required anisotropic component. We do so using a two-step uncertainty evaluation strategy that does not rely on multiple samples of data to capture the DU of a patient’s gross tumour volume (GTV) or clinical target volume. For simplicity, we will just refer to the GTV in the following. First, the clinician delineates two contour sets; one which bounds all voxels believed to have a probability of belonging to the GTV of 1, while the second includes all voxels with a probability greater than 0. Next, one specifies a probability density function for the true GTV boundary position within the boundaries of the two contours. Finally, a patient-specific PTV, designed to account for all systematic errors, is created using this information along with measurements of the other systematic errors. Clinical examples indicate that our margin strategy can produce significantly smaller PTVs than the van Herk margin recipe. Our new radiotherapy target delineation concept allows DUs to be quantified by the clinician for each patient, leading to PTV margins that are tailored to each unique patient, thus paving the way to a greater personalisation of radiotherapy.

## 1. Introduction

In radiotherapy, dose distributions are designed for each patient with the aim of achieving acceptable probabilities of tumour control and normal-tissue toxicity. The target volumes consist of the gross tumour volume (GTV) and the clinical target volume (CTV), as defined by ICRU (ICRU 2010).

The planning target volume (PTV) accounts for the various geometrical uncertainties, which limit the accuracy and precision of delivering planned doses to tumour targets. The PTV is a geometrical concept, created by enlarging the CTV by a margin that is designed to ensure the CTV is covered by the intended dose over the course of treatment, with a predefined level of confidence.

Target volume delineation is a fundamental source of geometrical targeting uncertainty, often being a dominant contributor to the PTV (Njeh 2008, Thwaites 2013, Segedin and Petric 2016). The magnitude of delineation uncertainty (DU) is currently estimated by measuring variations between contours produced by different observers (BIR 2003, Tudor *et al* 2020). This inter-observer variability is reported in the literature for a range of tumour sites (Leunens *et al* 1993, Logue *et al* 1998, Meijer *et al* 2003, Weiss and Hess 2003, Song *et al* 2006, Li *et al* 2009, Persson *et al* 2011, Chung *et al* 2012, Feng *et al* 2012, Hellebust *et al* 2013, Petric *et al* 2013, Duane *et al* 2014, Peulen *et al* 2015, Seravalli *et al* 2015, Segedin and Petric 2016) and organs at risk (OARs) (Li *et al* 2009, Gay *et al* 2012, Sandström *et al* 2016). A limitation of current practice is that DU is only measured for samples of patients, which prevents margins from accounting for DU associated with each individual patient.

The importance of margin anisotropies were demonstrated for both targets and OARs (Meijer *et al* 2003, Bell *et al* 2016, Gurney-Champion *et al* 2017). However, a limitation in DU measurement and PTV growing tools provided by commercial Treatment Planning Systems is that margins can only account for uncertainties specified along three cardinal axes.

The common approach of estimating uncertainty via the statistical analysis of a series of measurements, such as delineations, is classified as a Type A evaluation of uncertainty (Kuyatt and C E 1994, JCGM 2008, 2012). An alternative method, referred to as Type B evaluation, is based on scientific judgement using ‘all the available relevant information on the variability’ of the quantity being measured (Kuyatt and C E 1994, JCGM 2008, 2012). Both methods generate a standard deviation (SD) as an estimate of the SD for a population. Type B uncertainty estimates can be as reliable as Type A, particularly when Type A evaluations are derived from small sample sizes (Kuyatt and C E 1994, JCGM 2008). Furthermore, Type A and Type B uncertainty estimates can be combined to give a combined uncertainty.

Our standard CTV-PTV margin is determined by the van Herk margin recipe (van Herk *et al* 2000), henceforth referred to as MvHMR. In its simplest form the isotropic PTV-margin m, in the presence of systematic and random uncertainties, expressed by SDs Σ and *σ* respectively, is given by equation (1) where *σ*_p_ is a measure of the penumbra width. The parameters α and ß determine the confidence level that the CTV is covered by a specified isodose for a given fraction of the patient population

\begin{eqnarray*}{m}=\,\alpha {\mathrm{\Sigma }}+\beta \sqrt{{\sigma }^{2}+{\sigma }_{{\mathrm{p}}}^{2}}-\beta {\sigma }_{{\mathrm{p}}}.\end{eqnarray*}

With respect to DU, the MvHMR is limited by two assumptions. First, it assumes that the patient population is sufficiently homogeneous such that Σ adequately represent the whole population even when measured in only a small sample of patients.

Second, the MvHMR assumes that all geometric uncertainties can be modelled by translations of the volume of interest (VOI). For target delineation, this implies that the clinician always delineates the target with the correct size and shape, but with errors simply being in its position. This is in conflict with the publications that show delineation error to be anisotropic, as described above. Therefore, the MvHMR is not designed to account for anisotropy which is known to exist for DU.

We propose to overcome these limitations by introducing a different strategy to account for DU based on a Type B uncertainty analysis. This concept does not rely on samples of patient populations whose targets are determined by a small number of clinicians. The key new feature is that the uncertainty is estimated by a single experienced observer, who delineates a finite boundary interval whose positions are assumed to be specified with a negligible uncertainty. The subsequent reduction of the boundary interval to a PTV is more versatile than standard margin recipes and allows an extended exploitation of personal patient images. It also accounts for any anisotropy in the DU. We want to note that the outlined new methodology can be applied for the assessment of DUs of GTV and/or CTV. For simplicity, we will just refer to the GTV in the following.

## 2. Method

### 2.1. Type B delineation method

In this section, we describe our Type B uncertainty analysis for the imprecisely known ‘true’ GTV (GTV_T_).

#### 2.1.1. Type B uncertainty evaluation

A Type B uncertainty evaluation of a measurand *x*, requires the following be determined or estimated based on scientific judgement using all the available information:

• Containment limits: limits on the variation of *x* (Castrup 2001).
• The probability density function (PDF), *φ*(*x*), that a value *x* within the containment limit coincides with the unknown true value.
• Containment probability: the probability that the true value can be found within the containment limit (Castrup 2001). We assume this to be 1, unless stated otherwise.

#### 2.1.2. Containment limits and containment probability

In this section, we describe how the containment limits and containment probability for the imprecisely known GTV are generated.

In order to cope with the uncertainty of the GTV_T_ boundaries, two target structures will be drawn. First, the clinician excludes all voxels within the patient image that certainly do not belong to the GTV_T_. This outer, maximal target volume, referred to as the *Outer GTV* (GTV_O_), includes all voxels *g* with a non-vanishing probability of belonging to GTV_T_. The second new target structure, encompassed by GTV_O_, is the *Inner GTV* (GTV_I_) which only includes voxels *g* that are considered to be part of GTV_T_ with certainty. GTV_I_ and GTV_O_ define the containment limits of GTV_T_. They divide the imaging information into three classes of voxels *g* according to their probability *Q* of belonging to GTV_T_ according to equation (2). We will refer to *Q* also as boundary probability. *Q* quantifies the chance of finding the boundary of GTV_T_ outside a given level set of *Q*

\begin{eqnarray*}{Q}\left({g}\in {{\mathrm{GTV}}}_{{\mathrm{T}}}\right)=\left\{\begin{array}{cc}1 &amp; {\mathrm{if}}\,{g}\in {{\mathrm{GTV}}}_{{\mathrm{I}}}\\ \in (0,1] &amp; {\mathrm{if}}\,{g}\in {{\mathrm{GTV}}}_{{\mathrm{O}}}\\ 0 &amp; {\mathrm{if}}\,{g}\notin {{\mathrm{GTV}}}_{{\mathrm{O}}}\end{array}\right.\,.\end{eqnarray*}

### 2.2. Boundary PDF

The quantification of the boundary probability *Q* within the containment limits involves two essential components. First, the clinician is asked to describe the anticipated distribution of uncertainties for locating the true GTV in the boundary interval. This is facilitated by selection of a PDF, *φ*, used to quantify *Q*(*g*) for each voxel *g*. Second, the numerical values of *φ* need to be derived from the surfaces of the containment limits GTV_I_ and GTV_O_.

#### 2.2.1. Generation of *φ* from the containment limits

**Figure 1.**
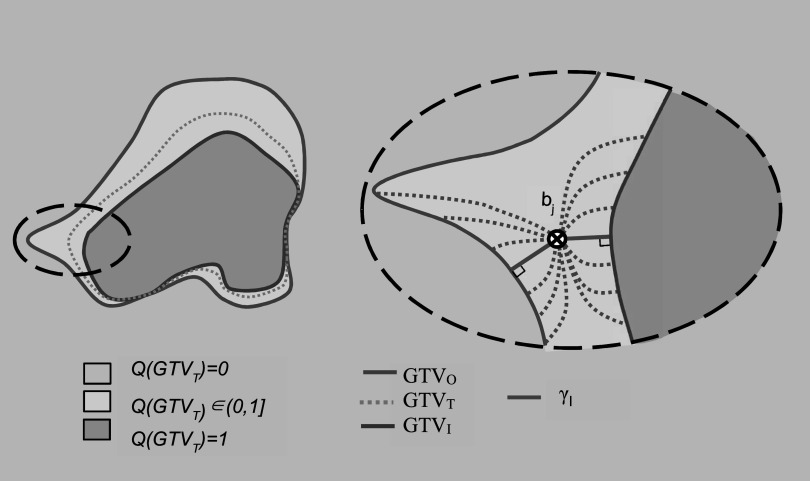
Left: an illustration of the containment limits and associated probabilities *Q*. Right, a close up illustration of the left dashed sector. Each dotted green curve illustrates a potential curve from the surface of GTV_I_ through the point b_j_ to the surface of GTV_O_. The shortest curve from GTV_I_ through b_j_ to GTV_O_, *γ*, is given by the solid green curve.

The boundary probability function *φ*, related to any voxel *g*, is defined on the shortest trajectory *γ*_g_ connecting the surfaces of GTV_I_ and GTV_O_ and passing through *g*, where *γ*_g_ remains bounded by GTV_I_ and GTV_O_ along its whole length, as illustrated in figure 1. The length w_g_ of *γ*_g_, referred to as the boundary width, consists of the sum of the minimal distances *d*_g,I_ and *d*_g,O_ of g from GTV_I_ and GTV_O_ respectively, measured whilst bounded by GTV_I_ and GTV_O_:

\begin{eqnarray*}{{w}}_{{\mathrm{g}}}={{d}}_{{\mathrm{g}},{\mathrm{I}}}+{{d}}_{{\mathrm{g}},{\mathrm{O}}}.\end{eqnarray*}

The PDF *φ* depends on *w*_g_ and the length, *d*_g_, of the trajectory measured from its starting point at GTV_I_, i.e. *φ* = *φ*(*w*_g_, *d*_g_). Its normalisation

\begin{eqnarray*}\displaystyle {\int }_{0}^{{{w}}_{{\mathrm{g}}}}{{\mathrm{d}}{d}}_{{\mathrm{g}}}\varphi ({{w}}_{{\mathrm{g}}},{{d}}_{{\mathrm{g}}})=1\end{eqnarray*}

states that for each trajectory, there will be one voxel belonging to the true boundary contour. While *w*_g_ introduces the absolute spatial scale for the specification of *φ*, we will work from now on with the relative distance *ρ* = *d*_g_/*w*_g_. The respective PDF *φ*_r_ (*ρ*) is related to *φ*(*w*_g_, *d*_g_) by

\begin{eqnarray*}{\varphi }_{{\mathrm{r}}}\left(\rho \right)={{w}}_{{\mathrm{g}}}\varphi \left({{w}}_{{\mathrm{g}}},{{w}}_{{\mathrm{g}}}\times \rho \right)\end{eqnarray*}

and satisfies the normalisation

\begin{eqnarray*}\displaystyle {\int }_{0}^{1}{\mathrm{d}}\rho \,{\varphi }_{{\mathrm{r}}}(\rho )=1.\end{eqnarray*}

#### 2.2.2. Selection of the PDF *φ*_r_

Now that the technical problem of defining the argument *ρ* of the PDF is addressed, we need to choose the detailed form of *φ*_r_. The freedom of selecting the general form of *φ*_r_ opens the opportunity for the clinician to critically evaluate the uncertainty information contained within the boundary interval. Depending on image quality, individual patient anatomy and a reflection on the drawing process of GTV_I_ and GTV_O_, the user can specify where they believe the true contour of GTV_T_ can be found. Many distributions exist that may suit this problem, however as a starting point, the following four distributions *φ*_r_, covering a practical and plausible spectrum of functions, are offered as a choice:

1. Uniform uncertainty
  This PDF indicates that the user assumes that any point within the boundary interval has the same chance of being part of the true contour, i.e.
  \begin{eqnarray*}{\varphi }_{r}\left(\rho \right)=1.\end{eqnarray*}
2. Linearly increasing from GTV_I_ to GTV_O_
  In this case, the user believes that the true contour is located closer to GTV_O_ and that voxels adjacent to GTV_I_ are located further away from the boundary of GTV_T_, i.e.
  \begin{eqnarray*}{\varphi }_{r}\left(\rho \right)=2\,\rho .\end{eqnarray*}
3. Linearly decreasing from GTV_I_ to GTV_O_
  The opposite bias, that GTV_T_ can be found closer to GTV_I_, is represented by
  \begin{eqnarray*}{\varphi }_{r}\left(\rho \right)=2(1-\rho ).\end{eqnarray*}
  The distributions of equations (8) and (9) are kept linear due to their simplicity.
4. Gaussian centred between GTV_I_ and GTV_O_

Finally, the user can also indicate that, according their judgement, the true contour can be found within the central region of the boundary interval. This is presented by the Gaussian curve, centred around *ρ* = 1/2 and takes the form

\begin{eqnarray*}{\varphi }_{r}\left(\rho \right)=\displaystyle \frac{1}{\sigma \sqrt{2\pi }}\exp \left(-\displaystyle \frac{{\left(\rho -\tfrac{1}{2}\right)}^{2}}{2{\sigma }^{2}}\right).\end{eqnarray*}

The width *σ* of the Gaussian determines the containment probability of the boundary interval, which is assumed to be close to 1. We chose *σ* = 1/6 leading to a containment probability of 0.997.

### 2.3. Creating the PTV

This section describes how to create a PTV using the information attained from the Type B delineation method above.

#### 2.3.1. Boundary probability maps and PTV generation

The cumulative boundary PDFs *C*(*ρ*), gives the probability of finding the GTV_T_ boundary inside a shell of a constant level of relative distance *ρ*. It is given by:

\begin{eqnarray*}{C}\left(\rho \right)=\displaystyle {\int }_{0}^{\rho }{\varphi }_{r}\left(\rho ^{\prime} \right){\mathrm{d}}\rho ^{\prime} .\end{eqnarray*}

Whilst the probability of finding the boundary outside a shell of constant *ρ* is given by *q*(*ρ*):

\begin{eqnarray*}{q}\left(\rho \right)=1-{C}\left(\rho \right).\end{eqnarray*}

The surfaces of GTV_I_/GTV_O_ refer to the largest/smallest shells with probabilities *q* = 1 and *q* = 0 respectively of finding the true boundary beyond these boundaries. The boundary probability *Q* is defined for each voxel according to equation (13) and can be visualized as boundary probability map within each patient

\begin{eqnarray*}{Q}\left({g}\in {{\mathrm{GTV}}}_{{\mathrm{T}}}\right)=\left\{\begin{array}{cc}1 &amp; {\mathrm{if}}\,{g}\in {{\mathrm{GTV}}}_{{\mathrm{I}}}\\ {\mathrm{q}}\left({\mathrm{\rho }}\right) &amp; {\mathrm{if}}\,{g}\in B\,\\ 0 &amp; {\mathrm{if}}\,{g}\notin {{\mathrm{GTV}}}_{{\mathrm{O}}}\end{array}\right..\end{eqnarray*}

#### 2.3.2. Coverage probability maps for DU

The accumulated pdf *C*(*ρ*) in equation (11) is used to create the PTV for DU. If the dose level *D*_T_ is prescribed as an iso-dose to the surface defined by the condition

**Figure 2.**
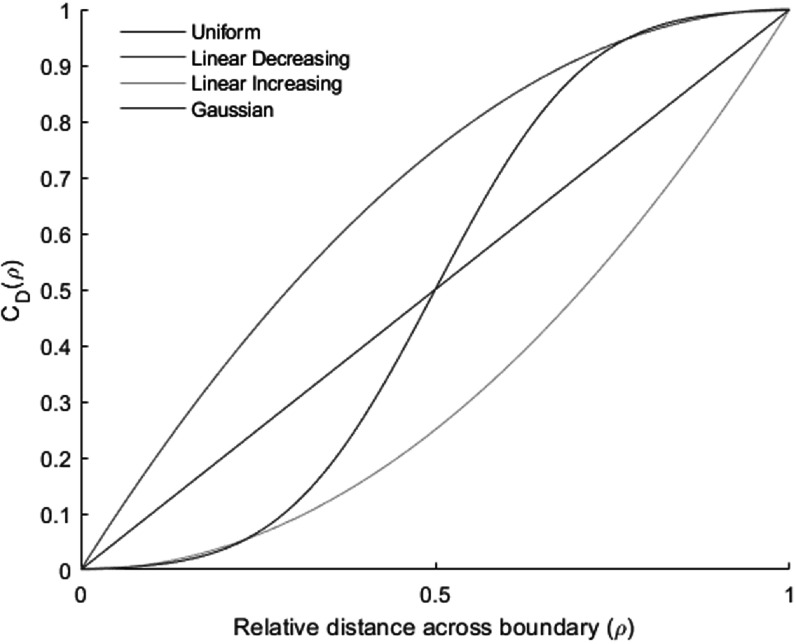
Coverage probability, *C*_D_, of covering GTV_T_ by the prescribed isodose as a function of the position of the shell in terms of relative distance, *ρ*.

\begin{eqnarray*}{{C}}_{{\mathrm{D}}}\left(\rho \right)={\rho }_{{C}}\,\end{eqnarray*}

then *C*_D_(*ρ*_c_) represents the coverage probability of the target for the prescribed dose level and is referred to as PTV_D_. The required value *ρ*_C_ depends on the selected coverage probability, *C*_D_, and the explicit form of the boundary PDF *φ*_r_(*ρ*). Figure 2 illustrates the relationship between the coverage probability and relative distance for the four specified boundary PDFs. The following equations define these relationships:

\begin{eqnarray*}\bullet \,{\mathrm{Uniform}}:\,{{C}}_{{\mathrm{D}}}\left(\rho \right)=\rho \end{eqnarray*}

\begin{eqnarray*}\,\bullet \,{\mathrm{Linear}}\,{\mathrm{Decreasing}}:\,{{C}}_{{\mathrm{D}}}\left(\rho \right)=2\rho -{\rho }^{2}\end{eqnarray*}

\begin{eqnarray*}\,\bullet \,{\mathrm{Linear}}\,{\mathrm{Increasing}}:\,{{C}}_{{\mathrm{D}}}\left(\rho \right)={\rho }^{2}\end{eqnarray*}

\begin{eqnarray*}\,\bullet \,{\mathrm{Gaussian}}:\,{{C}}_{{\mathrm{D}}}\left(\rho \right)\cong \,\displaystyle \frac{1}{2}\Space{0ex}{3.0ex}{0ex}1+{\mathrm{erf}}\,\Space{0ex}{3.0ex}{0ex}\displaystyle \frac{{{n}}_{{\mathrm{S}}}}{\sqrt{2}}\Space{0ex}{3.0ex}{0ex}\rho -\displaystyle \frac{1}{2}\Space{0ex}{3.0ex}{0ex})\Space{0ex}{3.0ex}{0ex})\Space{0ex}{3ex}{0ex}],\end{eqnarray*}

where the value *n*_s_ is the number of standard deviations assumed to be contained within the boundary width for the Gaussian distribution. Note that equation (18) is correct to within 0.5% for *n*_s_ ≥ 6.

#### 2.3.3. Incorporating the remaining systematic errors into the PTV

PTV_D_ presented above is designed to account for delineation error only, with a confidence level of *C*_D_. To account for all systematic errors, we follow the method proposed by Stroom *et al* (1999). Firstly, the boundary probability map, *Q*, is convolved with a probability distribution that describes the remaining systematic errors, resulting in a coverage probability map representing all systematic errors. The PTV corresponding to the desired coverage probability is given by the voxels bound by the corresponding level set on this coverage probability map. We refer to this final PTV as PTV_B_, since it is stems from Type B uncertainty evaluations and to distinguish it from the concept of the PTV.

#### 2.3.4. Clinical examples

Two clinical examples are presented to illustrate the differences in PTV_B_ arising from the different boundary PDFs, and the differences with respect to the PTV created using the MvHMR (PTV_MvH_). PTVs were created to give a 90% coverage probability for PTV_B_, and 90% confidence level for PTV_MvH_. The first case is a recurrent gynaecological cancer (RGC) GTV, and the second a prostate tumour. Typical cases were chosen based from a database selected for an extended clinical study yet to be published. Delineations were performed by clinicians highly experienced in treating these cases.

In these examples, we assumed the random errors to be negligible, i.e. to be 0. We assumed systematic errors, excluding delineation, to be 1.0 mm, based on data published by McNair *et al* for prostate treatments using fiducial markers and an online correction strategy (McNair *et al* 2008). PTV_MvH_ was grown from the GTV delineated in accordance with local clinical protocols (GTV_C_) for both examples.

The prostate example was delineated on CT alone. The delineation error used in the MvHMR was assumed to be 2.0 mm, based on data published by Alasti *et al* for the prostate delineated on CT (Alasti *et al* 2017).

The RGC example was delineated on co-registered CT and MRI. The delineation error Σ_D_ used in the MvHMR were those measured locally of 2.9 mm in the superior-inferior axes, 2.2 mm in the left-right axes and anterior-posterior axes. Although the MvHMR is not strictly designed to be used with varying margins, we do so to reflect common clinical practice.

## 3. Results

**Figure 3.**
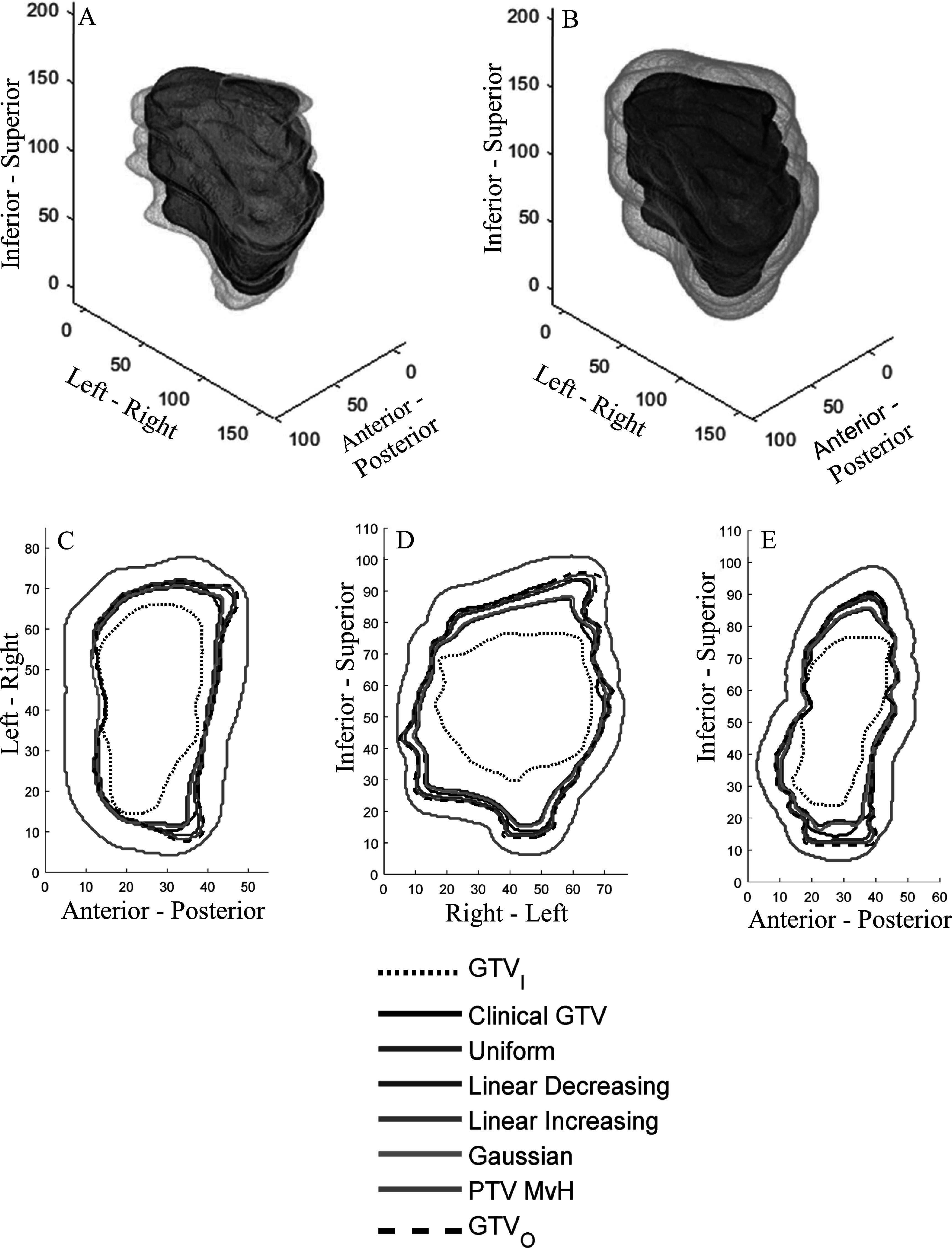
Clinical gynaecological example. (A) 3D rendering of GTV_I_ (yellow), the clinical GTV_C_ (black) and GTV_O_ (light blue). (B) 3D rendering of PTV_B_ (pink), using the Gaussian PDF, the clinical GTV_C_ (black) and PTV_MvH_ (green). (C)–(E) GTV_I_ and GTV_O_ contours, PTV_B_ and PTV_MvH_ boundaries through central axial, coronal and sagittal slices respectively. Axes are in mm.

**Figure 4.**
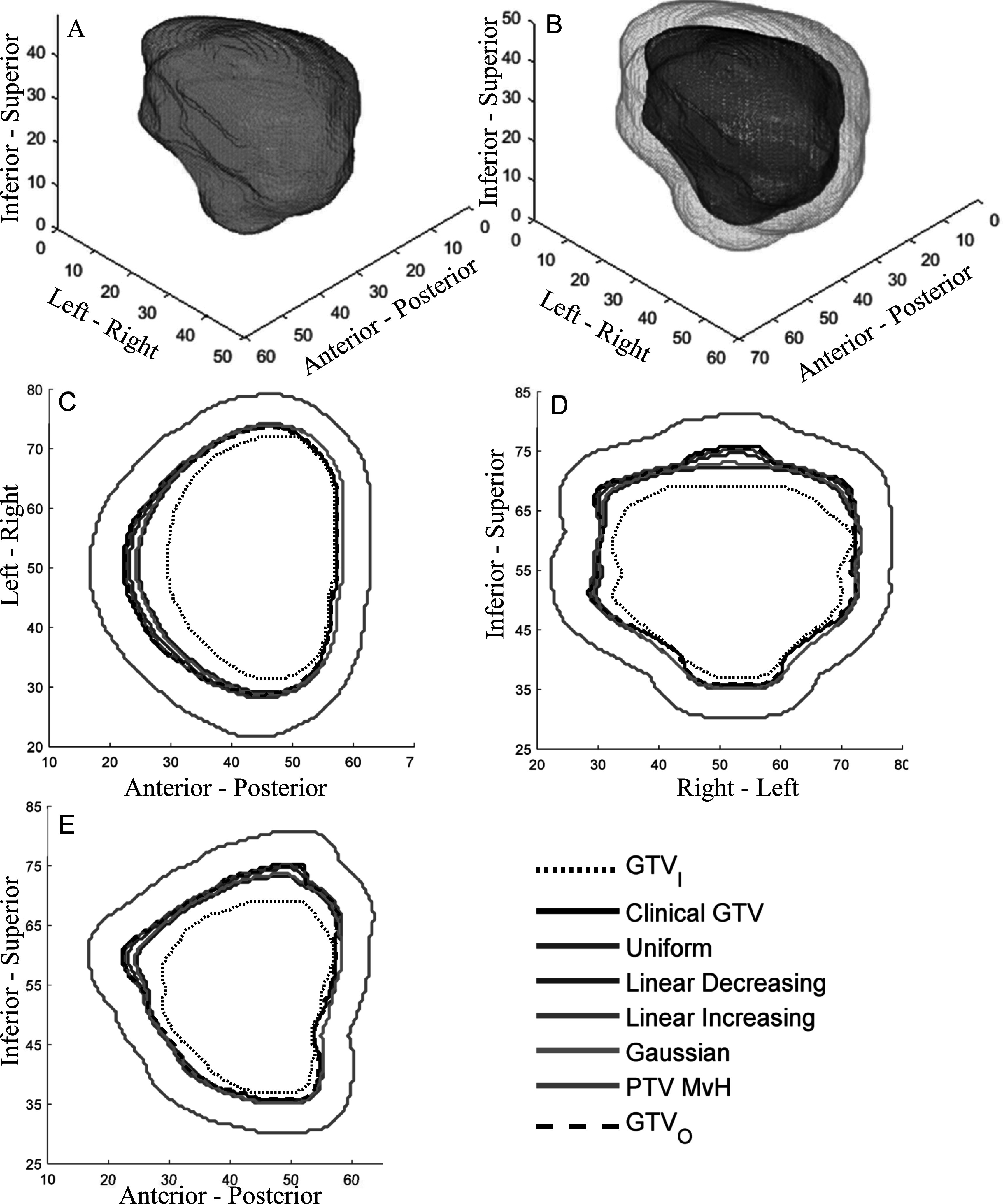
Clinical prostate example. (A) 3D rendering of GTV_I_ (yellow), the clinical GTV_C_ (black) and GTV_O_ (light blue). Note that GTV_C_ and GTV_O_ coincide over the majority of the surface. (B) 3D rendering of PTV_B_ (pink), using the Gaussian PDF, the clinical GTV_C_ (black) and PTV_MvH_ (green). (C)–(E) GTV_I_ and GTV_O_ contours, PTV_B_ and PTV_MvH_ boundaries through central axial, coronal and sagittal slices respectively. Axes are in mm.

Figures 3 and 4 show the outlines and PTVs for the RGC and prostate cases respectively. The gaps between GTV_I_ and GTV_O_ show that the clinicians had uncertainty when delineating these cases. It follows that the clinicians had to use their judgement to determine where to put the clinical GTV boundary given that uncertainty. The variation in the gaps demonstrates the uncertainty to be anisotropic. For the RGC case, the position of GTV_C_ varied with respect to GTV_I_ and GTV_O_. In contrast, for the prostate case, the clinician took a conservative approach to delineating GTV_C_ by making it the largest volume given all their uncertainties, and therefore it coincided with GTV_O_.

Figures 3(B)–(E) and 4(B)–(E) show that PTV_B_ is significantly smaller than PTV_MvH_ at all points in the target. These also show PTV_B_ to be more anisotropic due to the variation in its distances from GTV_C_.

Figures 3(C)–(E) and 4(C)–(E) also illustrate the different PTV_B_ boundary positions resulting from the different boundary PDFs. Naturally, we observe that the difference is negligible where the boundary width is small. As the boundary width increases, the uniform and linear increasing PTV_B_’s remain similar to each other, but become increasingly larger than the Gaussian and linear decreasing PTV_B_’s.

## 4. Discussion

In current practice, geometric uncertainties in radiotherapy are mitigated by adding PTV margins. These are based on estimates of the magnitude of each source of uncertainty, which are combined according to a margin recipe. The quality of each uncertainty estimate affects the appropriateness of the determined margin. The primary aim of this paper was to create PTVs that are based on the DU associated with each individual patient, as opposed to a sample of patients from a population, secondly to produce PTVs that reflect the anisotropy in the uncertainty, whilst thirdly, still accounting for all sources of systematic geometric uncertainties.

Population delineation error is currently estimated using a Type A approach, in which multiple clinicians delineate several cases. In this paper, we present a method for measuring delineation error based on Type B uncertainty evaluation methods. In this approach, the clinician uses their knowledge and experience to delineate the containment limits for the GTV (i.e. GTV_I_ and GTV_O_) and specify the PDF for the unknown ‘true’ GTV (i.e. GTV_T_) boundary based on where they believe the most likely true boundary position to be.

The effectiveness of the proposed method is dependent on the quality of the Type B uncertainty evaluation. Therefore, it is important that clinicians have the necessary knowledge and experience to delineate the containment limits. Even with this condition met, observer variability is likely to affect GTV_I_ and GTV_O_ delineations since delineating them is ultimately a subjective process. Prior to using the approach presented here, it is important that measures are implemented to; ensure clinicians have the necessary knowledge and experience, minimise any observer variability, and maximise consistency between clinicians. We hypothesise that measures used to reduce observer variability when standard delineation protocols are used, will also be effective in reducing any observer variability in the delineated containment limits and ultimately improve the quality of the Type B uncertainty evaluation. For example, interventions recommended by Chang *et al* (2017) and Vinod *et al* (2016b) could be developed and applied, these include; the use of atlases and guidelines, teaching for example through workshops, and peer review of outlines.

This Type B approach is proposed to address several limitations associated with using Type A methods for measuring delineation error in the clinic. The first limitation is its resource intensive nature; this arises from the need for multiple clinicians to delineate each case used for the uncertainty estimate. Performing studies in this way can also be logistically challenging due to the limited availability of clinicians. Limited resources restricts us to estimating delineation error on only a sample of patients. This prevents the creation of margins tailored to each individual patient. By using a Type B approach, a single clinician can estimate the delineation error for each patient, without relying on multiple clinicians. This approach would be less resource intensive than the Type A approach and so it may be feasible to do this for each and every patient in a clinical setting.

The second limitation the Type B delineation method aims to address is that DU is typically only measured along the cardinal axes, therefore, general information on the anisotropic nature of the uncertainty is lost. There are several studies in which anisotropic delineation error has been recorded (Remeijer *et al* 1999, Meijer *et al* 2003, Deurloo *et al* 2005, Peulen *et al* 2015, Bell *et al* 2016). However, these all rely on multiple observers making it unfeasible to do so routinely for each patient.

Some studies assume adequate spatial correlation in delineation error between patients in order to produce anisotropic PTVs (Nijkamp *et al* 2012, Bell *et al* 2016). This assumption is unlikely to be valid for all tumour sites, in such cases anisotropic population-based margins would be inappropriate. Xu *et al* (2015) present an alternative approach to estimating delineation error on a patient-by-patient basis for prostate cancer. To do this they proposed using the contours produced by a single observer, along with the contrast in the CT image, to model the DU. The authors then used coverage probability techniques to produce PTVs, which resulted in plans with improved target and/or OAR doses when compared with PTVs created using the MvHMR. One of the key limitations of their method is the reliance on a model to estimate DU, as opposed to using clinical data and clinician knowledge as done in this paper.

It is also not clear how DU would be modelled when multi-modality imaging is used for delineation, as is often the case in modern radiotherapy, or how well the model would translate to other tumour sites. These limitations will affect the produced PTVs. By measuring the uncertainty on a patient-by-patient basis, for example using the Type B method presented here, the DU can be measured anisotropically for each individual VOI by a single clinician regardless of anatomical site or imaging modalities used. The methods presented here could then be used to account for those uncertainties without also needing to make assumptions about the spatial correlations in Σ_D_ between patients. Furthermore, unlike the methods in this paper, Xu *et al* do not show how to account for other sources of systematic geometric uncertainties.

The third limitation the Type B delineation method aims to address is associated with the sample sizes used to measure delineation error. Inter-observer variability studies, which are used to measure delineation error, are generally limited by having a small number of observers. As described in the introduction, the uncertainty associated with a population standard deviation estimate depends on the sample size used, with the uncertainty decreasing with increasing sample size. For example, the authors of a review of 131 DU publications, showed them to have a median of 9 participants for GTV delineation error assessments (Vinod *et al* 2016a). The Chi-Square distribution shows that for a study with nine observers, the 95% confidence interval for the SD is 0.68 to 1.92 times the measured SD, which translates into an arguable significant uncertainty in the calculated PTV margin. The consequence of small sample sizes, and the reliance on sample data, means that Type B uncertainty evaluations can be as reliable as Type A evaluations (Kuyatt and C E 1994, JCGM 2008).

The approach of delineating the inner and outer limits of a target has been previously presented (Waschek *et al* 1997). In that paper, the authors used fuzzy logic to derive a PTV based on estimated impact of including different voxels within the PTV on tumour control and normal tissue complication probabilities. Using fuzzy logic to determine the PTV is conceptually very different to the geometric uncertainty based methods widely used, such as MvHMR, which is perhaps why such methods are rarely considered in clinical practice. The methods presented in this paper have the advantage that they are consistent with our standard geometric uncertainty based methods. Another advantage of the methods in this paper is that they account for all sources of systematic geometric uncertainty, unlike the fuzzy logic method.

Clinical examples presented to illustrate the methods and concepts, show that the clinician can establish regions of uncertainty using the method presented in this paper. They show that, unlike MvHMR, PTV_B_ mirrors the anisotropy in the DU. They show that, regardless of the boundary PDF used, PTV_B_ seems to be consistently smaller than PTV_MvH_. This reduction is a result of addressing the assumptions made in the MvHMR that are not valid for DU. The reduction presents the potential for dose escalation to the tumour and/or a reduction in toxicities by reducing the dose to surrounding tissue. However, as with any new technique, these methods should be assessed through a clinical study to ensure there are no unintended consequences from any reduction in the margin.

We have assumed that the selected PDF is appropriate for the whole VOI. This is a pragmatic assumption as defining multiple PDFs for one VOI would not be practical in a clinical setting, and may not result in a significant benefit. Four PDF options were presented to illustrate the methodology, however, there may be alternative appropriate distributions. The clinical examples show that where the boundary widths are narrow, the differences between the PTVs resulting from the different boundary PDFs are negligible. As the boundary width increases, the PTVs separate into two groups, with the uniform and linear increasing PDFs resulting in larger PTVs than the Gaussian and linear decreasing. These differences show that selecting an appropriate PDF can be important for larger boundary widths, as it can affect the PTV boundary position.

The method used to create PTV_D_ uses the concept of shells in a similar manner to Shusharina *et al* (2018), who used shells in place of a CTV and applied this concept for treatment optimization. Unfortunately, the authors do not show how these shells are derived in detail.

Where an expansion is required from the GTV to account for macroscopic spread, giving a CTV, any uncertainty in the expansion required will add to the overall uncertainty in the delineated target. In our approach, we do not aim to address the problem of uncertainty in GTV to CTV expansion. Instead, we rely on the clinician to consider it when delineating the containment limits for the CTV, i.e. CTV_I_ and CTV_O_. The CTV containment limits may be delineated directly, or by expanding GTV_I_ and GTV_O_ by the required GTV to CTV margin and modifying them according to anatomical boundaries if required. This approach is consistent with that taken in the MvHMR, in which uncertainty in the final CTV outline is considered, rather than any uncertainty in the GTV to CTV expansion. A possible future extension of the method present here would be to incorporate uncertainty in the GTV to CTV expansion and/or information on the distribution of microscopic disease around the GTV, for example as done by Stroom *et al* (2014) who developed a GTV to PTV margin.

## 5. Conclusions

A new concept for radiotherapy target delineation and how to design a corresponding PTV were presented to address several shortcomings of currently used margin recipes. The key innovative feature is that DUs are quantified by the clinician for each patient, leading to PTV margins that are tailored to the unique patient and the set of images representing the radiotherapy relevant anatomy, thus paving the way to a greater personalisation of radiotherapy. The two clinical examples considered seem to indicate that conventional margin strategies are less flexible and may be too conservative in ensuring dose coverage of the radiation target.
